# A novel cell-free mitochondrial fusion assay amenable for high-throughput screenings of fusion modulators

**DOI:** 10.1186/1741-7007-8-100

**Published:** 2010-07-26

**Authors:** Astrid C Schauss, Huiyan Huang, Seok-Yong Choi, Liqun Xu, Sébastien Soubeyrand, Patricia Bilodeau, Rodolfo Zunino, Peter Rippstein, Michael A Frohman, Heidi M McBride

**Affiliations:** 1 University of Ottawa Heart Institute, 40 Ruskin St., Ottawa, ON, USA; 2 Institute for Genetics and Cologne Excellence Cluster on Cellular Stress Responses in Aging-Associated Diseases (CECAD), Zülpicher Str 47, 50674 Köln, Germany; 3 Department of Pharmacology and the Center for Developmental Genetics, University Medical Center at Stony Brook, Stony Brook, NY 11794-5140, USA; 4 Department of Biomedical Sciences, Chonnam National University Medical School, Gwangju, Korea

## Abstract

**Background:**

Mitochondria are highly dynamic organelles whose morphology and position within the cell is tightly coupled to metabolic function. There is a limited list of essential proteins that regulate mitochondrial morphology and the mechanisms that govern mitochondrial dynamics are poorly understood. However, recent evidence indicates that the core machinery that governs mitochondrial dynamics is linked within complex intracellular signalling cascades, including apoptotic pathways, cell cycle transitions and nuclear factor kappa B activation. Given the emerging importance of mitochondrial plasticity in cell signalling pathways and metabolism, it is essential that we develop tools to quantitatively analyse the processes of fission and fusion. In terms of mitochondrial fusion, the field currently relies upon on semi-quantitative assays which, even under optimal conditions, are labour-intensive, low-throughput and require complex imaging techniques.

**Results:**

In order to overcome these technical limitations, we have developed a new, highly quantitative cell-free assay for mitochondrial fusion in mammalian cells. This assay system has allowed us to establish the energetic requirements for mitochondrial fusion. In addition, our data reveal a dependence on active protein phosphorylation for mitochondrial fusion, confirming emerging evidence that mitochondrial fusion is tightly integrated within the global cellular response to signaling events. Indeed, we have shown that cytosol derived from cells stimulated with different triggers either enhance or inhibit the cell-free fusion reaction.

**Conclusions:**

The adaptation of this system to high-throughput analysis will provide an unprecedented opportunity to identify and characterize novel regulatory factors. In addition, it provides a framework for a detailed mechanistic analysis of the process of mitochondrial fusion and the various axis of regulation that impinge upon this process in a wide range of cellular conditions.

See Commentary: http://www.biomedcentral.com/1741-7007/8/99

## Background

Mitochondria are highly dynamic organelles, whose plasticity allows them to respond quickly to cellular cues that regulate their cellular position, interconnectivity and function [1]. In this way, the dynamic behaviour of the mitochondria must be tightly coupled to cellular signalling cascades in order to respond in such a coordinated fashion. The molecular mechanisms that govern mitochondrial fusion have been best described using yeast genetics, where genome-wide screens have identified the essential components of the fusion reaction [2-4]. In addition to these genetic screens, a cell-free mitochondrial fusion assay has been developed using yeast mitochondria labelled with various fluorescent markers within the matrix and outer membranes [5]. This assay has provided important insights into the specific role of the fusion GTPases Fzo1 and Mgm1, along with the multispanning membrane protein Ugo1, in the regulation of mitochondrial docking, outer membrane and inner membrane fusion [5-7]. The yeast assay has, therefore, been critical in order to stage the reaction and follow mitochondrial docking and membrane fusion. However, this fluorescent output relies upon the direct user-based quantification of fused organelles using microscopy techniques, which has not been amenable to high-throughput screening and automation.

In contrast to the yeast model, one of the primary limitations in the study of mitochondrial dynamics in the mammalian system has been the reliance on semi-quantitative microscopic analysis of mitochondrial phenotypes within cells. Changes in mitochondrial morphology are quantified primarily using a subjective classification between 'fragmented' and 'fused'. These classifications cannot distinguish whether the observed phenotypes are the result of changes in fusion or fission (or both) and many cell types exhibit fused mitochondria under standard conditions, making quantification of morphology rather like examining spaghetti in a bowl. Two more quantitative approaches have been used to examine mitochondrial fusion, the polyethylene glycol induced whole cell fusion that allows the fusion between two differentially labelled populations of mitochondria [8,9], and the photoactivatible-GFP based assay, where small populations of mitochondria are fluorescently activated and their subsequent fusion events scored using fluorescent quantification [10,11]. Although superior to direct morphology analysis, these two methods are not very robust, and suffer from a number of technical issues. For example, the polyethylene glycol fusion itself probably affects the kinetics of mitochondrial fusion and the required addition of cyclohexamide is problematic for many applications. The PA-GFP assay takes many hours to obtain convincing results from just a single cell, making statistical analysis very tedious.

These technical issues make limited progress in the field of mitochondrial fusion, thus making it virtually impossible to identify and dissect the unique stages of the reaction, experiments which are routinely performed in other membrane fusion systems [12]. In addition, the inability to readily quantify mitochondrial fusion has made it difficult for scientists outside the field to investigate the contribution of mitochondrial fusion to multiple cellular processes. It is clear that mitochondrial dynamics plays an important role in many diseases, most notably Parkinson's Disease and other neurodegenerative conditions [13], as well as metabolic disorders such as diabetes [14]. The integration of scientists from these fields into the study of mitochondrial dynamics will depend upon the availability of robust assay systems to quantify mitochondrial fusion. Therefore, we have developed a new assay that uses a standard *Renilla *luciferase assay for the quantification of matrix-content mixing [15], providing a platform for scientists from diverse fields to examine the role of mitochondrial fusion in cell physiology and disease progression.

## Results and discussion

### Generation of a bimolecular complementation marker pair for the quantification of mitochondrial fusion

**Figure 1:**
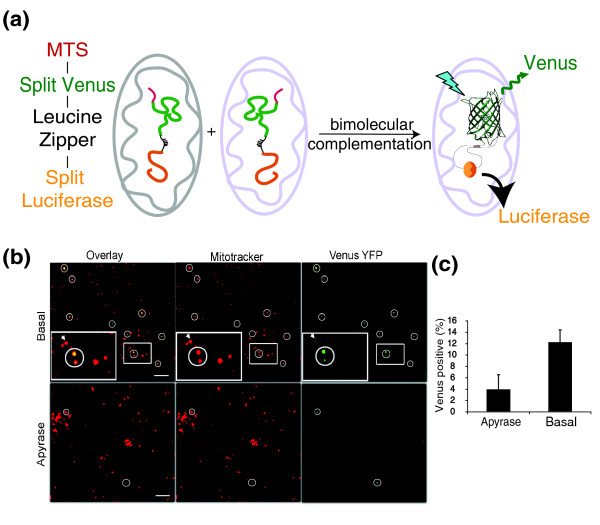
**Design of a bimolecular complementation assay to quantify mitochondrial fusion**. (a) Schematic representation of the bimolecular complementation strategy used in the development of the mitochondrial cell free fusion assay. (b) Isolated mitochondria were incubated in the presence of 5 mg/mL placental cytosol and energy regeneration mixture for 30 min at either 37° in the absence (top panels) or presence (bottom panels) of apyrase. Confocal images of each reaction revealed the presence of respiring mitochondria labelled with MitofluorRed633 (middle panels). Fused mitochondria show complementation of Venus yellow fluorescent protein (YFP; right panels). Insets in the top panels show a 2.4× magnification of the area represented within the boxed region. Circled regions show mitochondria positive for Venus YFP and the arrow within the inset highlights a fused mitochondrion beside one that has not complemented the Venus fluorescence. Representative images are shown, scale bars, 10 μ. A Gaussian blur filter of 1 pixel was applied to these images. (c) Quantification of (b) taken from 200-350 mitochondria per condition. The data are the average of two independent experiments.

In order to develop a high-throughput mitochondrial fusion assay, we adapted a bimolecular complementation strategy that would allow multiple outputs [15]. cDNAs were generated encoding mitochondrial targeted split Venus and R*enilla *luciferase separated by a leucine zipper. Fusion between mitochondria expressing each of these chimeric proteins would lead to their dimerization through the leucine zipper motifs, promoting the functional assembly of both luciferase and Venus yellow fluorescent protein (YFP) [16,17]. These constructs were designated N-MitoVZL (venus-zipper-luciferase) and C-MitoLZV (luciferase-zipper-venus; Figure 1a).

The constructs were used to generate two populations of stable suspension HeLa (sHeLa) cell lines, each expressing one of the split constructs. One litre of each stable line of sHeLa cells was grown to confluence and harvested by centrifugation and mitochondria were purified by differential centrifugation and flash frozen at -80°C.

**Figure 2:**
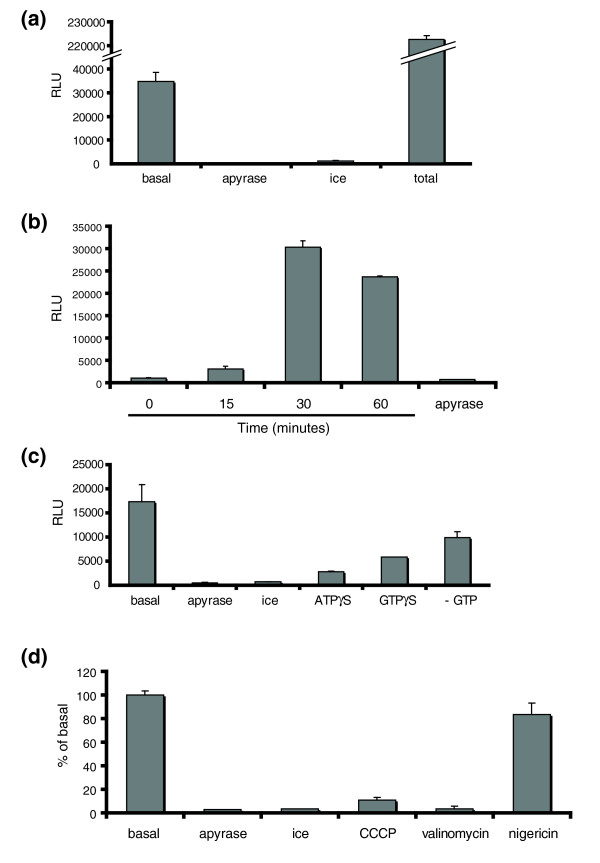
**Complementation of *Renilla *luciferase demonstrates the core requirements for mitochondrial fusion**. (a) The relative luciferase units (RLU) from the *Renilla *luciferase assay demonstrates that mitochondrial fusion requires physiological temperature and energy. Sonication of the basal fusion containing 5 mg/mL cytosol for 5 min, performed in the absence of trypsin, provided a maximum complementation of the reporter constructs to determine the efficiency of the reaction. Negative controls performed in the presence of apyrase or on ice are shown. The error bars in the histogram (as in all experiments) show the standard deviation of two independent reactions per condition. All conditions were repeated at least three times in duplicate. (b) Time course of the fusion reaction, as indicated. (c) Nucleotide dependence of the fusion reaction in the presence of 1 mM ATPγS, 1 mM GTPγS and in the absence of exogenous GTP, as indicated. (d) Mitochondrial fusion in the presence of 2 μM CCCP, 1 μM Valinomycin and 2 μM nigiricin demonstrates the dependence of the reaction on the charge gradient across the inner membrane.

As mitochondria are known to function quite well *in vitro*, most notably for metabolic and import reactions, we based the conditions of our assay on established buffering and energizing conditions [18,19]. Mitochondria of each population (50 μg each) were incubated with 5 mg/mL cytosol, GTP (0.5 mM) and an adenosine triphosphatase/succinate mixture, in a final volume of 25 μL. After mixing, the mitochondria were first concentrated by centrifugation and incubated on ice for 30 min. As reported in the yeast cell-free mitochondrial fusion assay, this step helps to facilitate mitochondrial docking [5], thereby enhancing the reaction. Following this step, the mitochondria were resuspended within the reaction buffer and incubated at 37°C for 30 min. Following the reaction, the extent of mitochondrial matrix content mixing could be detected by confocal or electron microscopy (Figure 1), or using a luciferase assay (Figure 2). For confocal imaging, the fusion reaction included MitofluorRed633 to label all of the respiring mitochondria. In order to quench potential background complementation that might occur due to the presence of ruptured mitochondria, 25 μg trypsin was added following the fusion reaction and incubated on ice for 20 min. This treatment also resulted in the dispersal of mitochondria, which helped to resolve the fused mitochondria by microscopy (not shown). The trypsin was then inactivated by incubation with soy bean trypsin inhibitor for a further 20 min on ice. Finally, the mixture was mounted on slides and imaged by confocal microscopy (Figure 1b). The mitochondria were first identified using the potentiometric dye and fused mitochondria, which represented 12% (+/- 2.4%) of the entire population, were observed through the visualization of the Venus YFP fluorescence (Figure 1b). These data reveal the complementation of Venus YFP within the matrix of fused mitochondria. In order to test whether mitochondrial fusion is nucleotide dependent, we added apyrase directly into the fusion reaction to deplete NTPs. This control reaction in the absence of energy revealed significantly fewer fused mitochondria within a field (4.0 +/- 2.0%).

**Figure 3:**
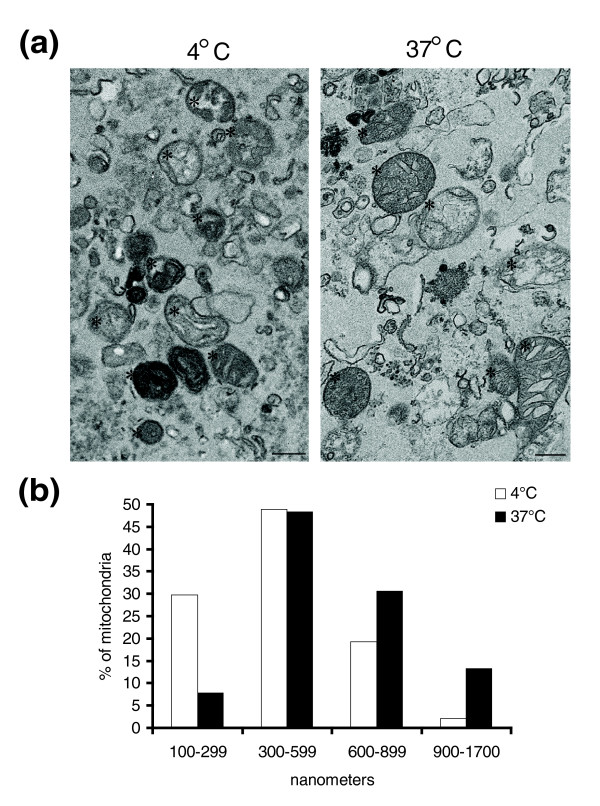
**Ultrastructural quantification of mitochondrial fusion *in vitro***. Fusion reactions performed in the absence of cytosol were scaled up to 50 μL and incubated either at 37° or 4°C. Mitochondria were re-isolated and embedded in low-melting agarose for electron microscopy analysis. (a) A representative section from the reaction incubated at 4°C (left panel), or 37°C (right panel). Asterisks point to intact, healthy mitochondria. Scale bars are 500 nm. (b) Quantification of the mitochondrial size, binned into 300 nm units. The data are representative of >300 mitochondria in each condition.

As a second output to quantify mitochondrial fusion, we decided to quantify any changes in the size of the mitochondria [5]. In order to examine this, we prepared fusion reactions performed at 4°, where docking should be facilitated, and reactions performed at 37°, for analysis by electron microscopy (EM). The trypsinization of the fusion reaction was omitted for this EM analysis to minimize the required steps. As can be seen in Figure 3a, the light membrane preparation of mitochondria used in our assays do contain other organelles. Western blot analysis showed that this preparation was highly enriched for mitochondria (anti-TOM20) and there was an absence of contaminating cytosol as indicated by the loss of tubulin signal (Additional File 1: Figure S1). Analysis of other organelle markers revealed the preparation was devoid of early endosomes and golgi, but retained ER and lysosomal markers (Additional File 1: Figure S1). The mitochondria within the preparation were largely intact, as indicated by the asterisks shown in Figure 3a. The majority of the mitochondria in these preparations are circular, with over 80% having a length:width ratio of 1:1 (not shown). When incubated at 4°, the mitochondrial diameter averages 500 nm. This diameter is increased upon incubation at 37°, with organelles regularly spanning 1 to 1.5 microns (Figure 3b). We quantified the size of 300 mitochondria from a fusion reaction completed at 37°C or 4°C and binned them into four categories. The data show that upon incubation at 37°C, there is an increase in the pool of mitochondria greater than 600 nm, from 21% to 44% of organelles counted (Figure 3b). This number is higher than the 12% observed by confocal imaging, however it should be noted that the complementation of Venus YFP can only be seen with the fusion of the N-MitoVZL with C-MitoLZV, where any homotypic fusion events will not be scored. However, the EM analysis scores all mitochondria, which may explain the increased numbers of larger organelles.

### Mitochondrial fusion is dependent on nucleotide hydrolysis and requires a charge gradient across the inner membrane

The confocal imaging confirmed the complementation of the Venus fluorescent protein (Figure 1), and the EM analysis demonstrated an increase in overall mitochondrial length (Figure 3). While these two outputs confirm the specificity of the assay, our main objective was to utilize the *Renilla *luciferase activity to provide a robust and high throughput method to score for mitochondrial fusion, with a very high signal-to-noise ratio. Figure 2a shows a representative reaction, where the basal reaction, containing cytosol, GTP and adenosine triphosphate regenerating system results in a signal around 35,000 relative luciferase units (RLU). Since our assay conditions also support mitochondrial protein import [18,20], it was possible that the signal we obtained may be due to the import of any free, cytosolic marker into the complementary mitochondria. This was not the case since the incubation of N-MitoVZL with cytosols taken from cells expressing C-MitoLZV, or visa versa, did not result in any signal (Additional File 1: Figure S2).

The absolute value of RLU obtained in the assay depended primarily on two factors. First, each mitochondrial preparation had a different load of the labels. Second, and perhaps more importantly, over the course of this study it became apparent that the length of time the mitochondria were left on ice prior to starting the reaction could reduce the efficiency of fusion. The dependencies on temperature and energy were unaffected, but the total values would decrease. Therefore it was important to use the mitochondria within the first 30-60 minutes of thawing them out (data not shown).

In order to test whether mitochondrial fusion is nucleotide and temperature dependent, we again added 0.1 U/μL apyrase, and performed a reaction at 4°C. As expected, mitochondrial fusion was not supported in the absence of energy, with ~400 RLU. Similarly, mitochondrial fusion required physiological temperature, with a reaction performed on ice resulting in only ~1200 RLU (Figure 2a). These results establish that the enzymatic amplification of the signal provides a very clear signal-to-noise ratio, which is essential for a high-throughput design. In order to estimate the efficiency of mitochondrial fusion *in vitro*, we disrupted all of the mitochondria within the reaction using 5 min pulses of sonification to allow maximum complementation at 37°, independently of a fusion event. The trypsin step, which is required to remove any background complementation coming from broken mitochondria after the reaction, was omitted in this reaction. With this, we detected ~227,500 RLU. Given that the basal reaction in this experiment gave 35,000 counts, it indicates that approximately ~15% of mitochondria were fusing under basal conditions (Figure 2a). In all of the reactions performed with this system, the efficiency has been seen to range between 5%-20%, depending primarily on the mitochondrial preparation (not shown). This is similar to other cell free fusion assay systems, including the yeast mitochondrial fusion system [5,21,22]. As mentioned above, this number is likely an under-representation of the total efficiency since it only scores for 'heterotypic' fusion events.

In a time course to examine the kinetics of mitochondrial fusion, we observe a steady increase in the fusion signal in the first 30 min. However, longer incubation times led to a decrease in the fusion signal, possibly due to degradation of the marker pairs (Figure 2b). The addition of non-hydrolysable analogues ATPγS and GTPγS inhibited mitochondrial fusion, consistent with a requirement for nucleotide hydrolysis. Omission of exogenous GTP led to a twofold reduction in fusion, suggesting that endogenous GTP within the mitochondria is limited and required for mitochondrial fusion (Figure 2c).

In order to determine the effect of the membrane potential across the inner membrane on mitochondrial fusion, we added 2 μM of the protonophore CCCP (carbonylcyanide-3-chlorophenylhydrazone). Fusion was abolished (Figure 2d) which is in agreement with previous studies in intact cells [8,23] and the yeast cell-free mitochondrial fusion assay [5]. To further dissect the membrane potential requirements, we examined the effect of valinomycin (1 μM) and nigiricin (2 μM). Valinomycin is an electrogenic potassium ionophore which abolishes the charge gradient. Electroneutral potassium/proton ionophore nigiricin retains the charge, but ablates the proton gradient. Valinomycin diminished the fusion reaction by 97%, whereas nigiricin, even at higher concentrations, inhibited the reaction by only 27% (Figure 2d). We conclude that mammalian mitochondrial fusion *in vitro *requires the charge gradient across the mitochondrial inner membrane but is independent of the chemical gradient.

### Mitochondrial fusion is modulated by signalling pathways

**Figure 4:**
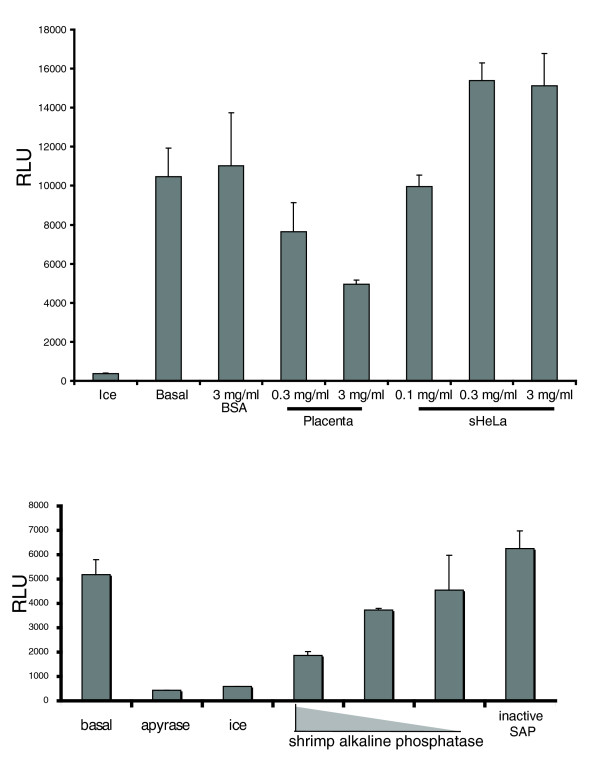
**Mitochondrial fusion is modulated by cytosol and phosphorylated proteins**. (a) Replacing cytosol with 3 mg/mL bovine serum albumin or 0.3 - 3 mg/mL placental or HeLa cytosol demonstrates a differential modulation of the fusion reaction depending upon the source. (b) Addition of 1, 0.1 and 0.001 units of shrimp intestinal phosphatase (SIP) show an absolute requirement for phosphorylated proteins in the fusion reaction. Heat inactivated SIP did not inhibit the reaction.

Similar to the yeast mitochondrial fusion reaction, the reaction was efficient in the absence of exogenous cytosol. Nonetheless, there was a profound consequence of cytosol addition, depending on the state of the cell at the moment of cytosol preparation. Figure 4a shows that the addition of 0.1, 0.3 and 3.0 mg/mL (final) cytosol prepared from non-confluent sHeLa cells undergoing normal proliferation stimulated the reaction in a concentration-dependent manner. However, the addition of 0.3 and 3 mg/mL purified placenta cytosol led to an inhibition of mitochondrial fusion in a dose-dependent manner. This cytosol inhibition was not due to osmotic effects, since 3 mg/mL BSA (bovine serum albumin) did not influence the reaction (Figure 4a). The results demonstrate that the amount of mitochondrial fusion can be modulated by cytosolic factors either in a negative or positive way depending on the source of cytosol. In this way, although we have efficient fusion in the absence of cytosol, the efficiency of the reaction is cytosol-dependent. One potential difference between placenta and sHeLa may be the status of signalling pathways that are activated upon the isolation of cytosol. In to test whether phosphoproteins may be required for mitochondrial fusion, we added Shrimp Alkaline Phosphatase (SAP) into the reaction. The data show that the dephosphorylation of proteins leads to a profound and concentration dependent inhibition of the reaction (Figure 4b). Heat-inactivated phosphatase was not inhibitory (Figure 4b).

**Figure 5:**
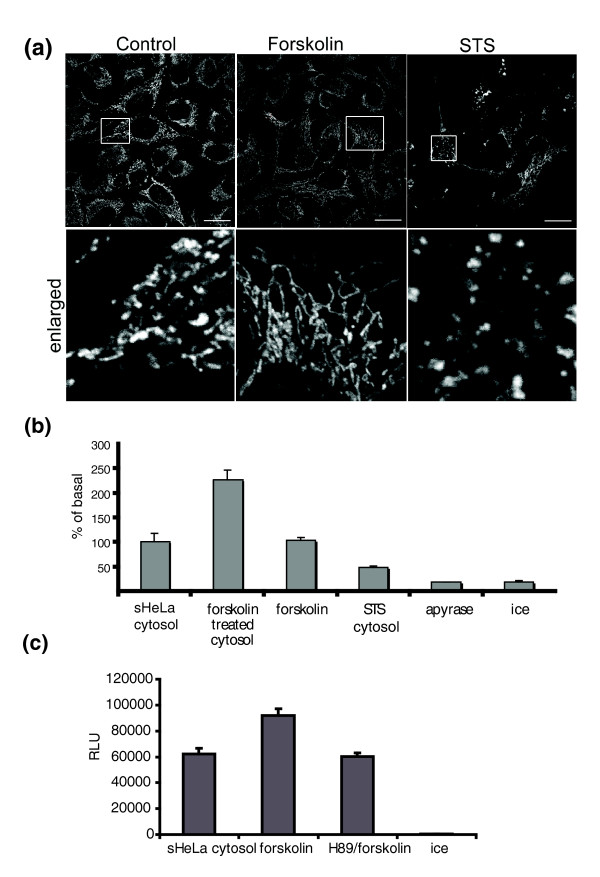
**Signal transduction cascades regulate the mitochondrial fusion reaction**. (a) HeLa cells were either untreated (left panels), treated with 10 μM forskolin for 30 min or with 1.5 μM staurosporin for 4 h were fixed and stained with anti-prdx3 antibodies to label mitochondria. Bottom panels show a 3× magnification of the boxed areas. Scale bars are 10 μ. (b) *Renilla *luciferase complementation is shown for mitochondrial fusion reactions containing 5 mg/mL sHeLa cytosol, or cytosols treated with forskolin or staurosporin, as indicated. The addition of 10 μM forskolin directly to the fusion reaction does not have any effect. Apyrase and 4°C controls are also shown. (b) As in b, including a control where HeLa cells were incubated with 10 μM forskolin for 30 min in the presence of its inhibitor H89 (10 μM, 1 h pre-incubation) prior to harvesting the cytosol for addition into the cell free assay.

In order to further probe the impact of signalling pathways on the fusion reaction, we isolated cytosols from drug-treated sHeLa cells to activate different pathways. We treated sHeLa cells with staurosporin (STS, 1.5 μM) for 6 h to induce apoptosis, collected the apoptotic cytosol by ultracentrifugation and added it back into the fusion reaction. As expected from previous studies [10,24], and the microscopic analysis of treated cells (Figure 5a), apoptotic cytosol inhibited the fusion reaction (Figure 5b). This demonstrates how cytosolic factors can modulate mammalian mitochondrial fusion. In contrast to the inhibition of fusion during cell death, it has been shown that the activation of protein kinase A (PKA) through the cAMP pathway leads to the phosphorylation of DRP1 and an inhibition of mitochondrial fission, leading to a more connected mitochondrial reticulum [25-27]. Upon addition of 10 μM forskolin to HeLa cells, we also observed an increased interconnectivity of the mitochondrial reticulum within the first 30 minutes (Figure 5a). In order to test whether this phenotype may reflect an increase in fusion that may accompany DRP1 inhibition, we harvested cytosol from forskolin-treated sHeLa cells and added this to the cell-free fusion reaction. Relative to control cytosol, forskolin-treated cytosol led to a 1.5 - to twofold stimulation in mitochondrial fusion (Figure 5b and 5c). Addition of forskolin directly into the reaction did not have any effect on the efficiency of fusion (Figure 5c). Further incriminating the adenylate cyclase pathway in promoting fusion, stimulation by forskolin could be prevented by a prior incubation with the PKA inhibitor H89 (1 h, 10 μM; Figure 5c). This confirms that forskolin acts through the established plasma membrane-bound adenylate cyclase whose downstream signalling mechanisms converge on the mitochondria to activate mitochondrial fusion.

## Conclusion

Our understanding of both the regulation and the mechanisms of mitochondrial fusion in mammalian cells has been limited by the tools available to systematically dissect the process at the biochemical level. We have here provided the first automated assay design to quantify mammalian mitochondrial fusion *in vitro*. Our data show that the reaction is physiologically controlled, requiring energy, temperature and the mitochondrial inner membrane potential. The fact that our mitochondria are fusogenic in the absence of exogenous cytosol, and the mitochondrial preparations are relatively free of cytosol (Additional File 1: Figure S1), suggests that the core machinery that drives mitochondrial fusion is primarily associated with the mitochondria. However, the modulation of the reaction by the addition of exogenous cytosol shows that the reaction is ultimately cytosol-dependent. The strength of the *Renilla *luciferase signals make this assay perfectly suited for screening factors required for fusion. However, the limitations of this assay are that we do not differentiate between the early steps of mitochondrial tethering, docking and the mixing of matrix content. The Nunnari laboratory has already optimized confocal and EM approaches that stage the reaction at each step of docking, outer membrane fusion, inner membrane fusion and matrix content mixing [5-7]. However, that approach remains very labour intensive. Future assay development could see the use of these marker pairs on outer membrane or intermembrane space proteins, which could then allow us to harness the luciferase-based output in evaluating each step of mitochondrial fusion.

The evidence that mitochondrial fusion is coupled to cell signalling pathways has been primarily correlative with little knowledge of the molecular mechanisms that underlie these links [1]. Using our cell-free assay system, we have provided direct evidence that shows how mitochondrial fusion is tightly regulated by cytosolic signaling cascades. The stimulation of fusion upon activation of adenylate cyclase indicates that the fusion machinery is integrated within the metabolic status of the cell. It has been shown previously that cAMP regulates the phosphorylation of DRP1 [25-27], leading to the inhibition of fission activity. Here, our data further demonstrate that activation of PKA also stimulates fusion. This is a first step in dissecting the substrates for PKA that trigger an increase in fusion. Interestingly, the absolute inhibition of mitochondrial fusion upon the global dephosphorylation of proteins with SAP also confirms the importance of phosphorylation cascades in the regulation of mitochondrial dynamics, providing an opportunity to screen for kinases and phosphatases that play key roles in this regulation.

Finally, the addition of apoptotic cytosol to the fusion reaction leads to an inhibition of mitochondrial fusion (Figure 4b). It is known that mitochondrial fission is activated upon cell death, concomitant with a block in mitochondrial fusion [10,24]. Our assay system confirmed this result, further validating this method. More importantly, the cell-free fusion assay can now be used to help define the molecular requirements for the apoptotic inhibition of fusion. In general terms, the adaptation of this assay to high throughput screens, and the fractionation of cytosols to identify novel fusion factors, will provide an essential and universal tool for the advancement of this field.

## Methods

### Reagents

All chemicals were purchased from Sigma Aldrich (ON, Canada) unless indicated. ATP*S and GTP*S were obtained from Jena Biosciences (Jena, Germany). Culture media, fetal bovine serum and non-essential amino acids were from Invitrogen (ON, Canada). Anti-prdx3 antibodies were previously described [28]. N-MitoVZL and C-MitoLZV were used as described (Huang *et al. *, in revision).

### Generation of stable sHeLa cell lines

The plasmids encoding N-MitoVZL or C-MitoLZV were transfected separately into sHeLa cells using Lipofectamine 2000 (Invitrogen, ON, Canada) and the plasmids left to express for 48 h before adding 2 μg/mL puromycin for 2-3 weeks to select for stably expressing cells. Total populations were amplified and stored using standard protocols.

### Mitochondrial purification and storage

Stable suspension HeLa lines expressing either N-MitoVZL or C-MitoLZV were thawed and grown in 3 × 15 mm dishes with DMEM medium [with 10% fetal bovine serum (FBS), L-glutamate, non-essential amino acids and 2 μg/mL puromycin] before they were harvested and transferred to 2 L flasks where they were grown in suspension in sMEM (with 10% FBS, L-glutamate, non-essential amino acids and 2 μg/mL puromycin). After 1 L of each culture reached 8 × 10^5 ^-1 × 10^6 ^cells/mL, the cells were harvested by 20 min centrifugation at 3000 × g at 4°C. Keeping all steps at 4°C, the pellets were washed in mitochondrial isolation buffer [MIB; 220 mM Mannitol, 68 mM sucrose, 80 mM KCL, 0.5 mM EGTA, 2 mM Mg(CH_3_COO)_2_, 10 mM HEPES pH 7.4, sterile filtered and freshly added protease inhibitors (Roche Diagnostics, Quebec, Canada) and 2 mg/mL fatty acid free BSA] and resuspended in 1.5× volumes in chilled MIB and the cells were ruptured with a glass dounce homogenizer. The homogenate was centrifuged for 10 min at 3000 × g at 4°C and the post-nuclear supernatant (PNS) was isolated. In order to recover mitochondria that remained trapped in the nuclear pellet, the pellet was resuspended again in MIB, centrifuged at 3000 × g and the PNS was recovered. Mitochondria were harvested from the PNS by centrifugation of 9000 × g for 15 min at 4°C. The pellet was washed once in MIB and recentrifuged at 9000 × g. Afterwards, mitochondria were resuspended in MIB (lacking BSA) containing 10% glycerol, aliquoted, snap frozen in liquid nitrogen and stored at -80°C. The protein concentration was measured with a standard Bradford assay.

### Isolation of cytosols

sHeLa cells were grown either on 15 cm dishes in DMEM medium (including non-essential amino acids, glutamate and 10% FBS) or in 500mL suspension with sMEM media (including non-essential amino acids, L-glutamate and 10% FBS). For untreated cytosol, cells in suspension were harvested after reaching a density of 8 × 10^5 ^- 1 × 10^6 ^cells/mL. Cells grown in suspension were directly harvested by centrifugation.

For the preparation of staurosporin treated cytosol, the culture was incubated with 1.5 μM STS for 6 h, whereas forskolin treated cells were incubated with 10 μM forskolin for 30 min before harvesting. In order to harvest adherent sHeLa cells, the plates were washed with phosphate buffered saline (PBS), scraped in MIB and centrifuged for 1 min at 3000 × g at 4°C. The isolated cell pellets were washed in MIB and resuspended in 1× volume MIB buffer in order to achieve a highly concentrated cytosol. The cells were homogenized with a glass dounce homogenizer and the cytosol was cleared by ultracentrifugation for 40 min at 120,000 × g at 4°C. The supernatant containing cleared cytosol was aliquoted, snap frozen in liquid nitrogen and stored at -80°C. The protein concentration was measured with the Bradford method.

For the isolation of human placental cytosol, fresh placenta was minced and washed extensively in phosphate buffered saline. Washed tissue was homogenized using a Waring blender, in 2× volume of MIB. The post-nuclear supernatant was collected from a 3000 × g centrifugation step for 20 min at 4°C, and the mitochondria were removed by centrifugation at 9000 × g for 25 min at 4°C. The post mitochondrial supernatant was cleared of other organelles by ultracentrifugation for 40 min at 120,000 × g at 4°C. The cleared placenta cytosol was dialyzed twice at 4°C in fusion buffer [35 mM sucrose, 75 mM KCl, 0.5 mM EGTA, 2 mM Mg(CH_3_COO)_2_, 10 mM HEPES, pH 7.4] before it was aliquoted, snap frozen in liquid nitrogen and stored at -80°C. To determine the protein concentration, the Bradford assay was used.

### *In Vitro *fusion reaction

The *in vitro *fusion reaction was carried out in 1.5mL eppendorf tubes. Mitochondria of each population (50 μg each) were added into the reaction with 5mg/mL cytosol, where the final concentrations of reagents in the reaction were: 10 mM HEPES pH 7.4, 110 mM Mannitol, 68 mM sucrose, 80 mM KCL, 0.5 mM EGTA, 2 mM Mg(CH_3_COO)_2_, 0.5 mM GTP, 2 mM K_2_HPO_4_, 1 mM ATP(K^+^), 0.08 mM ADP, 5 mM Na succinate and 1 mM DTT. Mitochondria were concentrated by centrifugation at 9000 × g for 1 min and incubated on ice for 30 min. The mixture was then resuspended and warmed to 37°C for 30 min. Following the reaction, any signal arising from broken mitochondria was quenched with 25 μg trypsin on ice for 20 min. Then, trypsin was inactivated with 500 μg soy bean trypsin inhibitor for an additional 20 min on ice.

For the *Renilla *luciferase assays, the trypsinized mitochondria were concentrated by centrifugation at 9000 × g for 1 min and solubilised with 50 μL of 1× lysis buffer (Promega, WI, USA) on ice for 1 h. Complementation of *Renilla *luciferase is temperature dependent and does not form during the solubilization step. The lysate was placed in 96 well plates where the coelenterazine substrate was injected directly into the reaction in a Glomax Luminometer (Promega, WI, USA), following manufacturer's instructions. The bioluminescence photon counts were detected over an integral of 10 s and the data were analysed with Excel.

### Confocal imaging

For the imaging of the Venus complementation, 100 nM MitoFluorRed633 (Invitrogen, ON, Canada) was added into the fusion reaction in order to visualize the total respiring mitochondria. Following trypsinization of the fusion reaction, the mixture was mixed with 1× volume of 1.2% low-melt agarose, ensuring that the agarose was below 35°. This was immediately placed onto a glass slide and covered with a coverslip to disperse the mitochondria evenly. The samples were imaged using an Olympus IX80 FV1000 confocal microscope, with a 100× NA1.4 objective lens, with the 488nm and 633nm laser lines to excite the Venus and potentiometric dye MitofluorRed633, respectively. All images were obtained using sequential image acquisition under identical conditions and were processed using even thresholding settings. The figures were assembled in Adobe Photoshop; we applied a 1 pixel Gaussian blur filter within Photoshop to better define the mitochondrial signal relative to the noise.

### Electron microscopy

Following the mitochondrial fusion reaction, mitochondria were concentrated by centrifugation at 9000 × g for 1 min and resuspended directly in 1.6% glutaraldehyde in PBS pH 7.4 for fixation for 1 h at 4°C. The fixed mitochondrial pellet was then resuspended in 15%BSA in PBS containing 1.2% low melt agarose, recentrifuged and the resulting pellet cut into 1 mm pieces. The samples were then postfixed in 1% osmium tetroxide in 0.1 M na cacodylate buffer, *en bloc *stained in 3% aqueous uranyl acetate, dehydrated in ascending ethanol and embedding in Spurr epoxy resin. Ultra thin sections were cut on a Reichert Ultracut E ultramicrotome. Digital images were taken using a JEOL 1230 TEM at 60 kV adapted with a 2000 by 2000 pixel bottom mount CCD digital camera (Hamamatsu, Japan) and AMT software.

## Abbreviations

BSA: bovine serum albumin; cAMP: cyclic adenosine monophosphate; CCCP: carbonylcyanide-3-chlorophenylhydrazone; EM: electron microscopy; ER: endoplasmic reticulum; FBS: fetal bovine serum; MIB: mitochondrial isolation buffer; PBS: phosphate buffered saline; PKA: protein kinase A; PNS: post-nuclear supernatant; RFU: relative luciferase units; SAP: staurosporin; YFP: yellow fluorescent protein.

## Authors' contributions

ACS adapted the assay to the split luciferase/venus technology, performed most of the experiments and manuscript preparation. HH and SYC developed the N-MitoVZL and C-MitoLZV constructs used to develop the assay. LX performed immunofluorescence and EM experiments and contributed to the data presented in Figure 5. PB and RZ were instrumental in the early development of the cell-free fusion assay using alternative approaches that were later adapted to the split luciferase system. PR prepared the EM samples and related technical assistance. MAF directed the development of the N-MitoVZL and C-MitoLZV constructs and assisted with the writing of the manuscript. HMM directed the experiments and the writing of the manuscript.

## Supplementary Material

Additional file 1 **Supplemental figures S1 and S2**. Supplementary figures. Click here for file
