# Supplementary material for: A novel cell-free mitochondrial fusion assay amenable for high-throughput screenings of fusion modulators
Source: BMC Biol. 2010 Jul 26;8:100. doi: 10.1186/1741-7007-8-100 (PMC2919466; doi:10.1186/1741-7007-8-100)
Supplement: Additional file 1 — Supplemental figures S1 and S2. Supplementary figures. [file 1741-7007-8-100-S1.PDF]

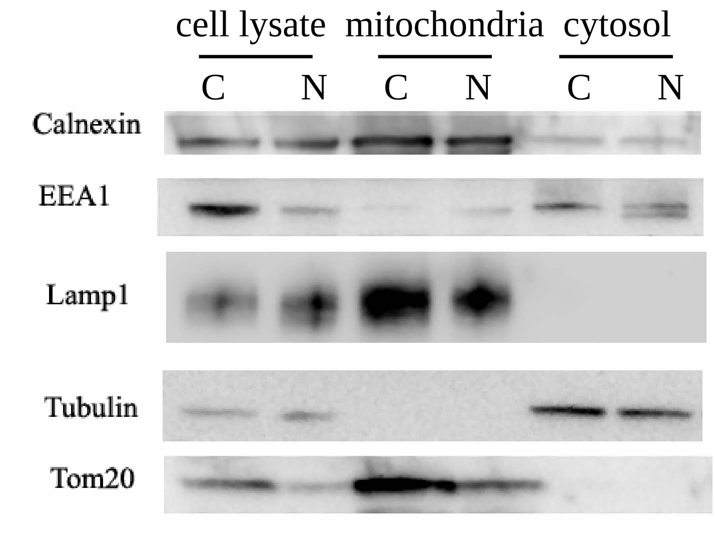

Supplemental figure S1. Characterization of the mitochondrial preparations by western blot. A representative analysis is shown. C and N: cytosol derived C-MitoLZV or N-MitoVZL, respectively. Indicated extracts (30 μg of protein) were analyzed for protein markers by western blot as indicated. Calnexin (SPA-860, Stressgen): ER; EEA1(610457, BD Biosciences): endosome; Lamp1 (SC-5570, Santa Cruz Biotechnology): lyzosome; Tubulin (T3526, Sigma): cytosol; Tom20 (612278, BD Biosciences): mitochondria.

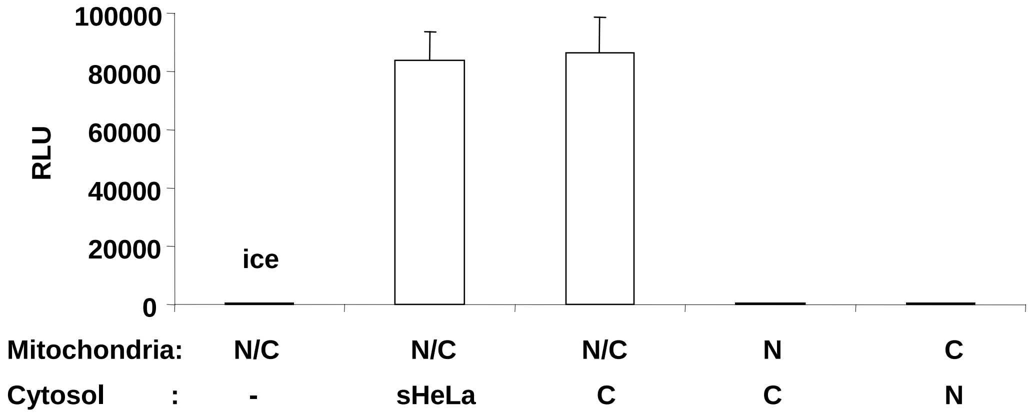

Supplemental figure S2. No measurable import associated with the fusion assay. Fusion assays of mitochondria isolated from N-mitoVZL (N) and/or C-MitoLZV-transfected cytosols (C) were performed in the presence of cytosols obtained from untransfected sHeLa (sHeLa), N-mitoVZL or C-MitoLZV-transfected sHeLa cells, as indicated. All assays were performed at 37 degrees except for the 4 degrees (ice) reaction. All cytosols were used at 3 mg/ml final protein concentration.
